# Assessment of antiretroviral treatment (ART) care service provision in Tigray Region health centers, North Ethiopia

**DOI:** 10.1186/s12913-015-1032-8

**Published:** 2015-09-10

**Authors:** Shewaye Belay Tessema, Mesafint Molla Adane

**Affiliations:** Institute of Biomedical Sciences, College of Health Sciences; Mekele University, Mekelle, Ethiopia; Department of Public Health, College of Medicine and Health Sciences, Bahir Dar University, Bahir Dar, Ethiopia

## Abstract

**Background:**

Client satisfaction is a vital component and main concern intertwined with strategic decisions in service provisions. To improve efficiency of services, eliciting the opinion of users about the available services and identifying factors associated with dissatisfaction is very critical. Thus, the main objective of this study was to assess the perceived levels of clients’ satisfaction with health services at ART clinic level in health centres of Tigray Region in Ethiopia.

**Methods:**

Cross sectional study was conducted from May to June 2013 in Tigray Region ART clinics. A total of 714 ART care user were included in the study using both purposive and probability sampling technique. Data was collected by using structured questionnaire and the collected data was analysed using Statistical Package for the Social Sciences (SPSS) version 16.0. Crude and Adjusted logistic regression analyses were carried out to identify the associated factors underlying perceived levels of clients’ overall satisfaction. Finally, the results were presented with table as well as odds ratio (OR) and 95 % confidence interval (CI).

**Results:**

A total of 714 study participants were enrolled in this study. An overall satisfaction level of 89.6 % was reported by ART care service users. Higher scores of satisfaction of services provisions were reported for courtesy and respect (95.80 %) followed by privacy (93.28 %). On the other hand, respondents’ dissatisfaction was rated 35.32 % for toilet cleanliness followed by 26.19 % for availability of additional drugs. As for overall satisfaction and associated factors, adjusted logistic regression analyses showed that marital status [AOR = 2.01 (95 % CI: 1.11, 3.60)], educational status [AOR = 3.13 (95 % CI: 1.15, 8.53)], travel distance to reach health centre [AOR = 3.59 (95 % CI: 1.23, 10.50)], toilet cleanliness [AOR = 2.22 (95 % CI :1.62, 6.32)], and ART drug availability [AOR = 2.60 (95 % CI :1.18, 6.52)] were found to have influence on overall ART service satisfaction status.

**Conclusions:**

This study revealed high level of client satisfaction rate and were associated with preventable and modifiable factors such as marital status, educational status, travel distance to reach health centre, toilet cleanliness and ART drug availability. Therefore, countermeasures such as increasing access to ART service, availing clean toilet and ART drugs may further increase client satisfaction level in the region.

## Background

Human immunodeficiency virus (HIV) infection, a worldwide phenomenon, is a serious public health challenge. HIV infection has globally claimed over 20 million lives, and currently over 34 million people carry the infection [1]. Even though AIDS remains a global pandemic, Ethiopia is one of the highly affected Sub-Saharan countries [2].

Ethiopian Demographic and Health Survey of 2011 [3] indicated an HIV prevalence of 1.5; which differs geographically: urban adult HIV prevalence was 4.2 % while rural adult HIV prevalence was 0.6 %. ANC results also document wide variations among urban settings in different parts of the country. According to EDHS (2011) [4] estimates there are nearly 789,900 people currently living with HIV/AIDS (607,700 adults and 182,200 children aged 0–14 years); and 952,700 AIDS orphans.

In Tigray Region, there are an estimated 56,900 people currently living with HIV/AIDS (PWHA). Based on Federal HIV and AIDS Prevention and Control Office (FHAPCO) current HIV prevalence report, of the PLHA in Tigray Region, 43,600 are adults above the age of 15 years and 13,300 are children (0–14 years old) [5].

Providing community health services in general and ART care services in particular is fundamental in alleviating morbidity, mortality and disease transmissions. Effective service delivery for patients is one of the strategies for alleviating infant and maternal mortality. This implies the necessity of providing efficient and comprehensive quality health service at all levels, especially in ART care health centres. Quality involves the consistent delivery of a product or service according to expected standards [6].

Quality assessment studies usually measure one of the three aspects: of service structure, process and outcome. Asking for and understanding users’ views and measuring patient expectations are seen as key components of both process and outcome evaluations. Effectiveness of health care services usually is determined by consumer satisfaction with the services provided [7]. Moreover, patient satisfaction is also directly related with utilization rate and hence meeting patient satisfaction improves the utilization of health services.

According to some reports, patients’ perspective on quality service is categorized into three components: managerial, professional and client based. Managerial quality is concerned with the cost effective use of resources, while the professional dimension of quality is concerned with clinical effectiveness and services setting standards of service utilizing systems. Patients’ satisfaction is a “patient cantered” process measure. It reflects the patients’ personal responses to, and evaluation of care [8]. Perceptions of poor quality of health service may, in fact, dissuade patients from using the available services because health concerns are among the most salient of human concerns. If the system cannot be trusted to guarantee a threshold level of quality, it will remain underutilized, be bypassed, used only for minor sickness, or used as a measure of last option [9].

To ascertain comprehensive quality ART care services and others for client’s satisfaction, Ethiopia’s Federal Ministry of Health (FMoH) has been leading a sector-wide reform effort aimed at significantly improving the quality and accessibility of health services at all levels of the country. As part of this reform, health facilities throughout the country have been streamlining their operational processes and building their capacities with a view to make their services more effective and efficient [10]. Thus, the main objective of this study was to assess the perceived levels of clients’ satisfaction with health services at ART clinic level in health centres of Tigray Region/Ethiopia.

## Methods

### Study design

Institution based cross-sectional study was conducted using quantitative data collection method at ART clinics in health centres of Tigray Region, Northern Ethiopia.

### Study area and period

Ten health canters found in five zones in Tigray region/Ethiopia were included in this study; namely, Western Zone (Adi-Goshu health centre & May Kadra health canter), South-Eastern zone (Hewane health centre & Adigudom health centre), Eastern zone (Wukro health centre & Adigrat health canter), Central zone (Enticho health centre & WukroMaray health centre) & Southern zone (Timuga health canter & Mekoni health centre). The study was conducted from May to June, 2013.

### Source and study population

The source population for this study was all people living with HIV (PWHA) and the study population were all ART care service beneficiaries in Tigray region/ Ethiopia.

### Inclusion and exclusion criteria

Potential participants were eligible if they were ART care beneficiaries, age of greater than 18 years and voluntary to participate. Critically ill patients and non-Ethiopians were excluded from this study. Admitted patients were also excluded as they necessarily passed through the out-patient units of the health centres.

### Variables

Independent variables: Socio demographic characteristics (address, age, sex, marital status, occupation and educational status)

Dependent variable: Overall satisfaction status

### Operational definitions

#### Overall satisfaction status

based on the five points Likert scaled 50 items analysis result, ART service clients who responded very dissatisfied, dissatisfied and neutral were classified as dissatisfied while those who responded satisfied and very satisfied were classified as satisfied.

### Sample size determination

The estimated sample size for the study was determined using EPI Info ver. 7 (at confidence level (1-α) =95 %, power (1-β) =80 %, an assumption of 50 % frequency Expected with confidence limit of 5 % and a design effect of 1.7. Assuming the five zones in Tigray serve as clusters, each cluster hold 164 participants, the total sample size became (131 subjects times 5 zones) gives 656 and adding 10 % for non response (66);accordingly, the estimated final total sample size was 721.

### Sampling technique and procedures

All the five zones and respective health centres in Tigray Region were assumed to be the same with respect to the study topic, because their staffing pattern and administration structures are all typical for Tigray Region context. To increase representativeness in terms of ART service accessibility, the research team employed purposive sampling to select 10 health centres by including all zones of the region. Then, the already determined sample size (721) was proportionally allocated to each health centres based on their ART service beneficiaries’ population size. Finally, ART service users were selected using systematic sampling method.

### Data collection procedures and instruments

The quantitative data were collected by interviewing ART service users using structured questionnaire by 7 senior nurses from Mekelle University after they had been trained for two days on data collection techniques by the principal investigators. Moreover, In addition to the primary investigators, 3 health research professional supervisors were employed as field supervisors after training.

### Data entry, management and statistical analysis

All data were entered into EPi Info ver. 7, cleaned and exported to Statistical Package for the Social Sciences (SPSS) version 16.0 software for analysis. In addition to the deceptive analysis, crude and adjusted logistic regression analyses were carried out to identify the associated factors underlying perceived levels of clients’ satisfaction. Finally, the results were presented in percentages as well as adjusted odds ratio (AOR) and 95 % confidence interval (CI).

### Ethical consideration

The ethical approval and clearance to undertake the research was obtained from the Research and Ethical committee (REC) of Mekelle University. An official permit letter was obtained from Tigray Regional Health Bureau and permissions were also sought from each district health offices and health centre. Finally, informed consent was obtained from clients after the purpose of the study explained to them.

## Results

The socio-demographic characteristics of clients are summarized in Table 1. A total of 714 study clients were interviewed making 99.03 % response rate. According to this study the number of females (63.45 %) was higher than the number of males. The mean age of respondents was 34.9 (± SD, 9.4) years ranging from18 to 75 and the majority (43.3 %) of clients lied in the age range of 25 – 34 years old. Significant number of clients (42.6 %) had not attended formal education, (32.5 %) were divorced, (25.2 %) were farmer by occupation and majority (70.3 %) of the study clients were urban dwellers.

**Table 1:** Socio –demographic characteristics of ART care users in North Ethiopia, 2013 (*n* = 714)

characteristics		Number	Percent
Sex	Male	261	36.55
	Female	453	63.45
Age	18–24	69	9.66
	25–34	309	43.28
	35–44	227	31.79
	>45	109	15.27
Marital status	Single	164	2322.97
	Married	187	26.19
	Divorced	232	32.49
	Widowed	131	18.35
Educational Status	No formal education	304	42.58
	Read & Write	76	10.64
	Grade 1–6	182	25.49
	Grade 7–12	131	18.35
	Diploma &>	21	2.94
Occupation	Government employee	72	10.08
	Merchant	136	19.05
	Farmer	180	25.21
	No job	149	20.87
	Daily labourer	146	20.45
	Defence force	31	4.34
Address	Urban	502	70.3
	Rural	212	29.7

This survey has shown that a total of 640 (89.6 %) ART care service users reported favourable ART service satisfaction level in the health centres of Tigray Region/Ethiopia.

The primary HIV screening places were government health institutions for 655 (91.74 %) and the rest 59 (8.26 %) were screened at private health institutions. The study participants gave different reasons for their first time HIV screening test such as 453 (63.45 %) because of sickness, 156 (21.85 %) to know status, 57 (7.98 %) for pregnancy test. Concerning frequency of visits made to the health centres, most of them 586 (82.19 %) visited health centre once per month.

In the present study, courtesy and respect of health care providers for the clients was the leading 684 (95.80 %) satisfaction level followed by privacy during examinations (using a private room/curtained or screened area) 666 (93.28 %) respondents. On the other hand, 172 (24.16 %) participants rated for delay to get health personnel at health centres, 15.241 % of them respond that they waited for 1–2 h to get health personnel.

Overall, 560 (91.80 %) managed to get the laboratory services in the health centres. From the 50 service beneficiaries sent to other health institutions for additional laboratory investigations, only 7 (14.00 %) were able to get the service in other governmental health institutions but the rest 43 (86.00 %) clients obtained the laboratory services from private health institutions. Of those 352 study participants who were prescribed additional drugs and supplies, 60.51 % have gotten the drugs from the health institution free of charge; the rest bought the drugs from private pharmacies. Overall, 187 (26.2 %) were dissatisfied with the unavailability of drugs in the health centre ART clinics.

Regarding the overall service provisions assessments of the health centres, ART clinics, nurses’ activities were evaluated in special assessment mechanisms. Accordingly, high satisfaction were found in aspects of: the amount of privacy nurses gave to the clients (91.32 %; nurses’ awareness of clients’ needs (89.90 %); how nurses listened to clients’ worries and concerns (89.92 %); the amount of time nurses spent with the clients (88.66 %%) and how often nurses checked to see if the clients were okay (89.08 %%) were among services rated for high clients satisfaction.

As for overall satisfaction and associated factors, our adjusted logistic regression analyses model showed that marital status [AOR = 2.01 (95 % CI: 1.11, 3.60)], educational status [AOR = 3.13 (95 % CI: 1.15, 8.53)], address/travel distance to reach health centre [AOR = 3.59 (95 % CI: 1.23, 10.50)], toilet cleanliness [AOR = 2.22 (95 % CI :1.62, 6.32)], and ART drug availability [AOR = 2.60 (95 % CI :1.18, 6.52)] were found to have influence on overall ART service satisfaction status among ART service users in Tigray Region/Ethiopia. (See Table 2).

**Table 2:** Adjusted Logistic Regression results of ART service satisfaction status and associated factors among ART care users in North Ethiopia, 2013. (*n* = 714)

Predictor variables		ART service satisfaction		Crude	Adjusted
				OR (95 % CI)	OR (95 % CI)
		Yes	No		
Sex	Male	232	29	0.88 (0.54, 1.45)	0.82 (0.47,1.42)
	Female	408	45	1.00	
Age	18–24	65	4	1.64 (0.49,5.46)	1.35 (0.36, 5.01)
	25–34	268	41	0.66 (0.32,1.37)	0.56 (0.25, 1.26)
	35–44	208	19	1.11 (0.50,2.47)	1.06 (0.45,2.48)
	>45	99	10	1.00	
Marital status	Single	150	14	1.18 (0.53,2.61)	2.01 (1.11,3.63)
	Married	165	22	0.83 (0.40,1.71)	1.25 (0.52, 3.02)
	Divorced	207	25	0.91 (0.45,1.85)	1.02 (0.47,2.19)
	Widowed	118	13	1.00	
Educational Status	Illiterate	268	36	0.78 (0.18,3.51)	3.13 (1.15,8.53)
	read & Write	63	13	0.51 (0.11,2.46)	0.44 (0.08,2.29)
	Grade 1–6	171	11	1.64 (0.34,7.94)	1.45 (0.28,7.80)
	Grade 7 – 12	119	12	1.04 (0.22,5.03)	0.97 (0.19,5.02)
	Diploma &>	19	2	1.00	
Occupation	G/ employee	93	10	0.99 (0.42,2.32)	0.87 (0.34,2.20)
	Merchant	116	20	0.62 (0.30,1.27)	0.66 (0.311.42)
	Farmer	158	22	0.76 (0.381.55)	0.82 (0.37,1.82)
	No job	141	8	1.87 (0.76,4.60)	1.42 (0.56,3.64)
	Daily labourer	132	14	1.00	
Address/travel	Urban	190	22	1 (0.59,1.70)	3.59 (1.23, 10.50)
	Rural	450	52	1.00	
Toilet cleanliness (*n* = 688)	Yes	415	30	3.06 (1.87,5.01)	2.22 (1.62, 6.32)
	No	199	44	1.00	
ART drug availability	Yes	480	47	1.72 (1.04,2.86)	2.60 (1.18,6.52)
	No	160	27	1.00	

There was a statistically significant association between ART service satisfaction status and marital status [AOR = 2.01 (95 % CI: 1.11, 3.60)], those ART service users who were singe in marital status were 2.01 times more likely to be satisfied with the ART service compared to ART service clients who were widowed.

Educational status also showed statistically significant association with ART service satisfaction status [AOR = 3.13 (95 % CI: 1.15, 8.53)]; ART service clients who were illiterate were 3.13 times more likely to be satisfied compared to clients who are diploma and above.

There was also a statistically significant association between ART service satisfaction status and address/travel distance to reach health centre [AOR = 3.59 (95 % CI: 1.23, 10.50)]; ART clients who came from Urban area were 3.59 times more likely to be satisfied compared to clients who came from rural area.

Similarly, health centre toilet cleanliness was also showed a statistically significant association with ART service satisfaction status [AOR = 2.22 (95 % CI :1.62, 6.32)]; ART service clients who had reported availability of clean toilet were 2.22 times more likely to be satisfied compared to clients who had reported invariability of clean toilet in the health centre.

In addition, there was a statistically significant association between ART service satisfaction status and ART drug availability [AOR = 2.60 (95 % CI: 1.18, 6.52)]; ART service clients who had reported availability of ART drug were 2.60 times more likely to be satisfied compared to clients who had reported invariability of ART drug in the health centre pharmacy.

## Discussion

This survey has shown that the overall satisfaction level of ART care service beneficiaries in the district health centres of Tigray Region was 89.6 %. This satisfaction level report is very high compared to different research reports from different areas [11]. On the other hand this finding is similar to other satisfaction surveys, for instance, a study conducted in Ethiopia at Jimma University Specialized Hospital on client satisfaction with anti-retroviral therapy services showed that most of the questions on level of satisfaction regarding the skill, attitude and interaction of ART staffs were answered positively [12]. The result can be attributed to different reasons; the foremost reason for this highest satisfaction rate in the present study could be due to the better attention given by the national and regional governmental higher officials and existence of non-governmental organizations specifically working on ART service such as Management Sciences for Health (MSH). Therefore, these better concerns and efforts could made fertile changes in the service delivery process of all health institutions including district health centres. Perhaps the other key reason for this high satisfaction result could be due to the current endeavours of building efficient health development army in Tigray region and in Ethiopia at large.

The majority (63.45 %) of the respondents in this study carried out HIV test because of sickness; indicating the fact that people are not using the opportunity to know their health-status ahead and care themselves before they become symptomatic cases of HIV/AIDS. T This finding is in agreement with a study conducted in Ethiopia that shows majority (88 %) of the respondents underwent HIV test due to illness [13]. The reason for not having an advance checkups and blood test for HIV could be fear of social stigma and discrimination [14]. Nevertheless, this is burning issue for the community in general as different incidences of HIV transmission scenarios could be there among communities including previously uninfected children and mothers.

In relation to primary HIV screening places of study participants, 91.74 % were screened in government health institutions and only 8.26 % of them in private health institutions. This indicates that HIV test was preferable in governmental health institutions than private or non-governmental health institutions. This could be explained by the availability services free of charge in the governmental health institutions. The other reason could be due to availability of better services in public health institutions than in private health service providers.

In the present study, 172 (24.16 %) participants reported 1–2 h delay to get ART service provider. This may indicate either there is large number of clients relative to the number of care provider staff members, or perhaps the other reason can be care provider staff members may not respect working hours in health centres.

In this study, additional laboratory procedures (excluding x-ray and the like) were ordered for (85.4 %) the clinic service users. Nevertheless, 50 (8.2 %) participants didn’t get the laboratory services in the health centres, where they usually benefited from and for free of charge; and 43 respondents got the laboratory services from private health institutions. This could be due to the nature of the laboratory procedures ordered to clients or unavailability of laboratory materials.

Access and cleanliness of the health centres latrines were the other aspects where satisfaction level was less compared to the other health service facets, 243 (35.32 %) of them were dissatisfied with cleanliness of toilets.

In addition to describing the overall satisfaction level of ART care service beneficiaries in the district health centres of Tigray Region/Ethiopia, the second main objective of this study was to assess the associated factors with overall satisfaction status, accordingly, this study showed that marital status [AOR = 2.01 (95 % CI: 1.11, 3.60)], educational status [AOR = 3.13 (95 % CI: 1.15, 8.53)], address/travel distance to reach health centre [AOR = 3.59 (95 % CI: 1.23, 10.50)], toilet cleanliness [AOR = 2.22 (95 % CI :1.62, 6.32)], and ART drug availability [AOR = 2.60 (95 % CI :1.18, 6.52)] were found to have influence on overall ART service satisfaction status among ART service users in Tigray Region/Ethiopia.

There was a statistically significant association between ART service satisfaction status and marital status [AOR = 2.01 (95 % CI: 1.11, 3.60)], those ART service users who were singe in marital status were 2.01 times more likely to be satisfied with the ART service compared to ART service clients who were widowed.

Moreover, educational status also showed statistically significant association with ART service satisfaction status [AOR = 3.13 (95 % CI: 1.15, 8.53)]; ART service clients who were illiterate were 3.13 times more likely to be satisfied compared to clients who are diploma and above. This is in agreement with the study in Jimma Hospital/Ethiopia in which satisfaction score was an in inverse relation to educational status that means high education was associated with low satisfaction score [15].

There was also a statistically significant association between ART service satisfaction status and address/travel distance to reach health centre [AOR = 3.59 (95 % CI: 1.23, 10.50)]; ART clients who came from Urban area were 3.59 times more likely to be satisfied compared to clients who came from rural area. This may be because of the long distance walk on foot for less than half an hour to get ART service.

Similarly, health centre toilet cleanliness was also showed a statistically significant association with ART service satisfaction status [AOR = 2.22 (95 % CI :1.62, 6.32)]; ART service clients who had reported availability of clean toilet were 2.22 times more likely to be satisfied compared to clients who had reported invariability of clean toilet in the health centre. This can be due to suitable environments such as clean toilets are required especially for HIV/AIDS patients to prevent different opportunistic and non-opportunistic co-infections.

In addition, there was a statistically significant association between ART service satisfaction status and ART drug availability [AOR = 2.60 (95 % CI: 1.18, 6.52)]; ART service clients who had reported availability of ART drug were 2.60 times more likely to be satisfied compared to clients who had reported invariability of ART drug in the health centre pharmacy. This can be due to incurring additional coast to buy the drugs from private pharmacies in which 39.49 % of ART users didn’t get drugs from the health centres. This finding is lower than study done by Girmay Adane (2006) in Mekelle regional referral hospitals, where about 61 % of those clients with prescription paper for drugs did not get the ordered drugs from the hospital pharmacies [16]. However, this finding is higher than a study conducted by Mesganaw and Getu (2003) in hospitals of the Amhara region, where about 27.2 % of the clients bought the prescribed drugs from private pharmacies [17].

## Conclusions

This study revealed high level of client satisfaction rate and were associated with preventable and modifiable factors such as marital status, educational status, travel distance to reach health centre, toilet cleanliness and ART drug availability. Therefore, countermeasures such as increasing access to ART service, availing clean toilet and ART drugs may further increase client satisfaction level in the region.

### Recommendations

As observed from this study, client satisfaction status was associated with preventable and modifiable factors. Therefore, health service managers, government officials and non-governmental organizations working in the region shall take countermeasures such as increasing access to ART service, availing clean toilet and ART drugs.
